# The Role of Carbon Nanoparticles as Lymph Node Tracers in Colorectal Cancer: A Systematic Review and Meta-Analysis

**DOI:** 10.3390/ijms242015293

**Published:** 2023-10-18

**Authors:** Georgios Koimtzis, Georgios Geropoulos, Leandros Stefanopoulos, Christopher Gwydion Chalklin, Ioannis Karniadakis, Vyron Alexandrou, Nikos Tteralli, Eliot Carrington-Windo, Andreas Papacharalampous, Kyriakos Psarras

**Affiliations:** 1 Department of General Surgery, University Hospital of Wales, Cardiff and Vale University Health Board, Cardiff CF14 4XW, UK; georgios.koimtzis@wales.nhs.uk; 2 Western General Hospital, NHS Lothian, Crewe Road South, Edinburgh EH4 2XU, UK; georgios.geropoulos@nhs.net; 3 Department of Electrical and Computer Engineering, Northwestern University, 633 Clark St, Evanston, IL 60208, USA; leandros@northwestern.edu; 4 Cardiff Transplant Unit, University Hospital of Wales, Cardiff and Vale University Health Board, Cardiff CF14 4XW, UK; christopher.chalklin@wales.nhs.uk (C.G.C.); ioannis.karniadakis@wales.nhs.uk (I.K.); 5 Urology Department, General Hospital of Thessaloniki “G. Gennimata-Agios Dimitrios”, Elenis Zografou 2, 54634 Thessaloniki, Greece; vyrwnal@hotmail.com; 6 Department of General Surgery, North Hampshire NHS Foundation Trust, Basingstoke RG24 9NA, UK; nikos.tteralli@hhft.nhs.uk; 7 Department of General Surgery, Grange University Hospital, Caerleon Road, Llanfrechfa, Cwmbran NP44 8YN, UK; eliot.carrington-windo@wales.nhs.uk; 8 Department of Surgery Larnaca General Hospital Pandoras, 6301 Larnaca, Cyprus; andreaspch96@hotmail.com; 9 Second Surgical Propedeutic Department, School of Medicine, Ippokrateio General Hospital, Aristotle University of Thessaloniki, Konstantinoupoleos 49, 54642 Thessaloniki, Greece

**Keywords:** colorectal cancer, lymph nodes, carbon nanoparticles

## Abstract

Colorectal malignancies are the third-most common malignancies worldwide, with a rising incidence. Surgery remains the treatment of choice and adequate lymph node dissection is required for accurate staging. The objective of this study is to assess the use of carbon nanoparticles in lymph node tracing and resection in cases of colorectal cancer. For that purpose, we conducted a systematic review and meta-analysis of studies included in Medline, Scopus, Embase, Cochrane Library, and Google Scholar databases. In the end, ten studies with a total number of 1418 patients were included in the final statistical analysis. The meta-analysis carried out showed that the use of carbon nanoparticles results in an increased number of lymph nodes harvested (WMD 6.15, 95% CI 4.14 to 8.16, *p* < 0.001) and a higher rate of cases with more than 12 lymph nodes harvested (OR 9.57, 95% CI 2.87 to 31.96, *p* = 0.0002). As a consequence, we suggest that carbon nanoparticles are used on a wider scale and that future research focuses on assessing the association between their use and overall patient survival. This study is limited by the fact that all included studies originate from China and by the fact that certain oncologic parameters and long-term outcomes have not been taken into account in the analysis.

## 1. Introduction

Colorectal cancer is the third-most frequent cancer and the second-most common malignant cause of death worldwide [1]. Based on recent predictions, the incidence of colorectal malignancies is expected to increase to 2.5 million new cases in 2035, while a worrying increase in the number of cases of colorectal cancer under the age of 50 has also been observed [2]. The most important risk factors that have consistently proven to have strong association are increasing age and male sex. Also, environmental and hereditary factors play an important role in the development of the disease, as positive family history has been identified in approximately 10–20% of patients diagnosed with colorectal cancer [2]. Colorectal cancer is mostly asymptomatic, but can also present with a large variety of symptoms and signs, such as rectal bleeding, anemia, change in bowel habits, abdominal pain, and bowel obstruction. The diagnosis of colorectal cancer is mainly established using colonoscopy. Nonetheless, only the diagnosis of advanced disease is relatively straightforward. Early colorectal malignancies may present as indistinctive mucosal lesions and therefore require careful inspection and optimal bowel preparation [2]. The 5-year overall survival rate for colorectal cancer patients is 64–67%, with an 89–90% 5-year survival rate in patients with early-stage cancer [3] that drops to 65–70% for stage III patients and further down to 14% for stage IV patients [4].

Based on the outcomes of various clinical trials, there seems to be large heterogeneity in the overall survival rate of the patients, and multiple clinical factors, such as the patients’ performance status, the level of tumor markers such as CA 19-9 and CEA, and the level of white cell count, hemoglobin, platelets, transaminases, and serum albumin, have been considered as prognostic factors without, however, a general consensus reached [5]. Nonetheless, surgery is the most efficient treatment for colorectal cancer and the pathological findings of the surgical specimen are the most decisive predictors of prognosis [6]. Since the lymph nodes are the most common site of metastasis of colorectal cancer, as many lymph nodes as possible should be retrieved during the operation [6]. Not only is the number of positive lymph nodes retrieved an important prognostic factor, but the total number of lymph nodes resected is another one as well, as it correlates with optimal mesenteric resection [6]. Based on the guidelines of the American Joint Committee on Cancer (AJCC) and the Union of International Cancer Control (UICC), the recommended minimum number of lymph nodes that needs to be included in the surgical specimen for accurate staging of nodal involvement is 12 [7]. However, this recommended number is often not achieved by traditional manual resection [7,8]. As a result, new technical methods are utilized in order to achieve a better lymph node harvest, such as the use of methylene blue [9] and the acetone elution and compression method [10].

Nowadays, nanotechnology is one of the most gallopingly progressing fields of science and has resulted in many achievements that are used in various fields of biology and medicine [11]. These applications have become achievable as a result of the production of nanoparticles, which are particles whose size is less than 100 nm. More specifically, nanotechnology has been constantly providing more nanomaterials to be used in cancer diagnosis and management [11,12]. Carbon nanoparticles are used in various fields, such as nanopharmacology, nanomedicine, and nanooncology, as they incorporate all the advantages of the nanotechnology industry, showing excellent absorption properties and the ability to aggregate [11]. Since the diagnosis and treatment of cancer are, at the moment, the most important problems in medicine, carbon nanoparticles have recently started to be used by surgeons in China for lymph node mapping in various operations carried out for malignancy, such as endometrial, breast, thyroid, gastric, and colorectal malignancies [12,13,14,15,16]. Carbon nanoparticles have been associated with an increased number of lymph nodes retrieved in surgery for the abovementioned malignancies, while they also improve the identification rate of small lymph nodes and can be used to trace sentinel lymph nodes in order to evaluate lymphatic metastasis [12,13]. Carbon nanoparticles have an average diameter of 150 nm and, as a result, can enter lymphatics that have a diameter of 120–500 nm and stain the lymph nodes black, but they cannot enter small blood capillaries (diameter of 20–50 nm), thus minimizing the adverse toxic effects [12,17]. However, carbon nanoparticles can alter the structure of specific proteins, potentially causing them to malfunction [11]. Ultimately, this can lead to the creation of inflammatory molecules as well as promotion of the body’s immune response, leading to cellular dysfunction. The time of administration of carbon nanoparticles in cases of colorectal cancer varies across different studies and the procedure can be carried out either weeks before the operation or the day before surgery, or even on the day of the operation either preoperatively or intraoperatively [16]. Nonetheless, most studies suggest that carbon nanoparticles should be injected around the tumor one day before the operation. Although the exact dose of carbon nanoparticles varies, care should be taken to avoid low dose as it may lead to insufficient dye of the lymph nodes, while an overdose may cause other problems. Thus far, experience has shown that 0.5 or 1 mL (25 or 50 mg) of carbon nanoparticle suspension, with or without dissolving it in appropriate normal saline, is adequate and suggested for identification of lymph nodes.

Currently, there are plenty of studies originating from single centers that confirm the advantages of carbon nanoparticles, and these findings have been corroborated by systematic reviews and meta-analyses, such as in the case of thyroid cancer surgery [12,18]. Nonetheless, regarding the use of carbon nanoparticles in colorectal cancer surgery, there is only one systematic review [16] with no quantitative synthesis and one meta-analysis [17] that has included only randomized control trials and advocates the need for further studies to establish the value of the technique.

The objective of this systematic review and meta-analysis is to investigate if the use of carbon nanoparticles as lymph node tracers in colorectal cancer leads to a higher number of lymph nodes harvested and a higher ratio of more than twelve lymph nodes harvested. The detection rate of metastatic lymph node dissection is also investigated in this study.

## 2. Results

**Figure 1:**
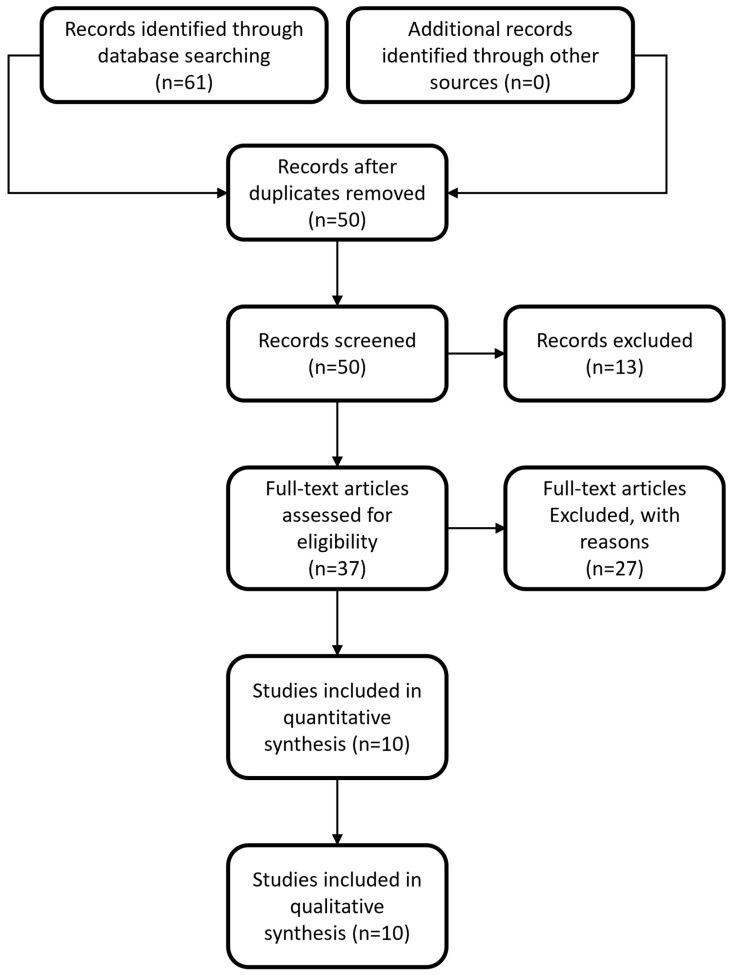
Flowchart depicting the selection process for inclusion of manuscripts in the article.

The initial search of the online literature resulted in a total number of 61 articles, while no other articles were identified by searching the current gray literature. Following removal of duplicates, the total number of articles was brought down to 50. These studies were screened based on their title and abstract and this process yielded 37 articles eligible for full-text analysis. Following full-text analysis, 27 articles were excluded since their methodology or their design/protocol did not investigate the same PICO question as the one investigated in our study, or because no full text was available, no control group was used, or data were insufficient. In the end, 10 articles [19,20,21,22,23,24,25,26,27,28] were included in the quantitative and qualitative analysis. The selection process flowchart of the studies is depicted in Figure 1.

The publication dates of the articles included in this study ranged from 2012 to 2021. The ten studies included in this meta-analysis had a total number of 1418, with 779 having received carbon nanoparticles while the rest were assigned to control groups. The basic information of the studies included in our study is portrayed in Table 1.

The meta-analysis of the data of the 10 included articles in this study revealed that the patients in the experimental group (carbon nanoparticle group) had a statistically significant higher number of lymph nodes harvested compared to the control group. The WMD was 6.15 (95% CI 4.14 to 8.16, *p* < 0.001). This analysis was performed using a random effects model, as data were heterogeneous (I^2^ = 93%, *p* < 0.001). This analysis is shown in Table 2. Moreover, the meta-analysis of the four studies [19,20,21,27] that provided data on the number of cases with more than twelve lymph nodes harvested showed that the carbon nanoparticle group had a statistically significant number of cases, with the required number of lymph nodes for accurate staging harvested (OR 9.57, CI 95% 2.87 to 31.96, *p* = 0.0002). The analysis of these data was also carried out using a random effects model since the data were also heterogeneous (I^2^ = 71%, *p* = 0.02). This outcome is portrayed in Table 3. Finally, the meta-analysis of the four studies [19,23,24,27] that provided data on the number of metastatic lymph nodes harvested showed that there was no difference between the experimental and the control groups (OR 1.14, CI 95% 0.87 to 1.49, *p* = 0.33). This analysis was also carried out with a random effects model due to the heterogeneity of the data (I^2^ = 66%, *p* = 0.03), and its outcomes are shown in Table 4.

The meta-regression analysis that was carried out due to the large heterogeneity of the data in all three analyses yielded values of I^2^ = 92.75%, 70.48%, and 65.85%, with respective *p* values of 0.000, 0.0172, and 0.0323, indicating the presence of residual heterogeneity in all of the analyses performed.

**Figure 2:**
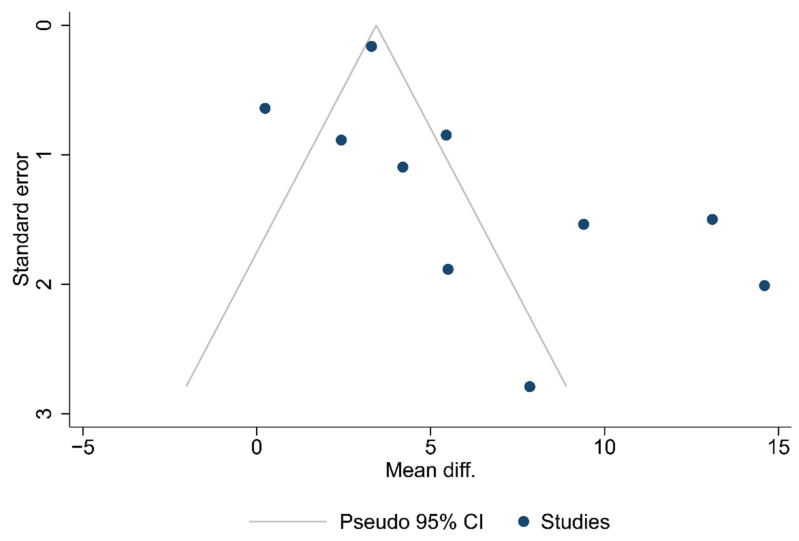
Funnel plot of the studies in the analysis of the total number of lymph nodes harvested.

**Figure 3:**
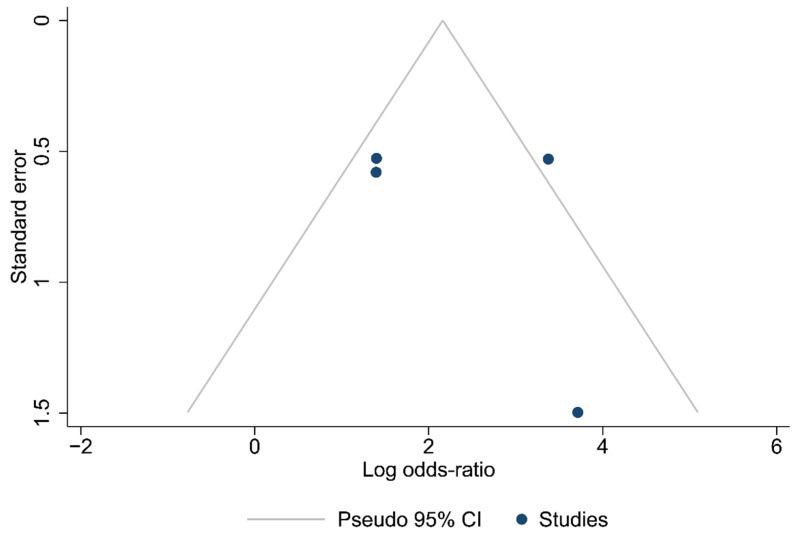
Funnel plot of the studies in the analysis of cases with ≥12 lymph nodes harvested.

**Figure 4:**
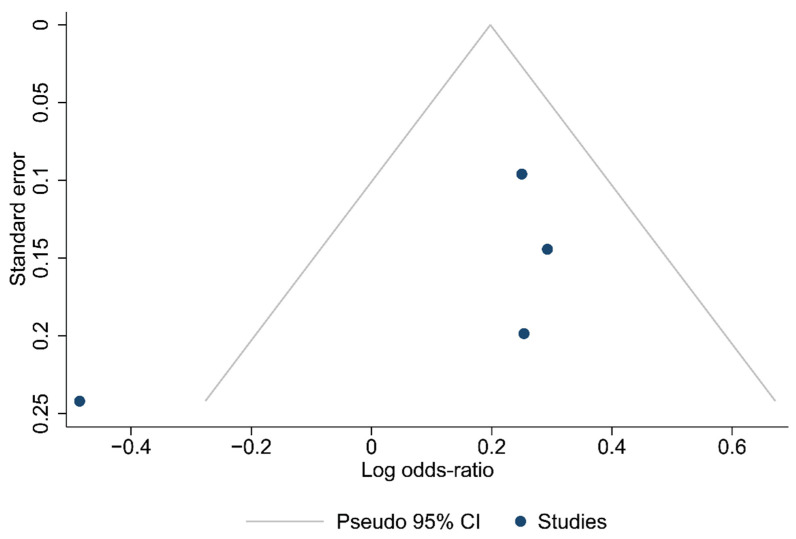
Funnel plot of the studies in the analysis of metastatic lymph nodes harvested.

Funnel plots for the articles that were included in each of the carried out analyses are shown in Figure 2, Figure 3 and Figure 4. According to the graphical presentations and the result of the Egger’s test, which yielded values of *p* = 0.0089, *p* = 0.1184, and *p* = 0.4521 for each of the analyses performed, respectively, publication bias is likely to be present only in the first analysis.

**Figure 5:**
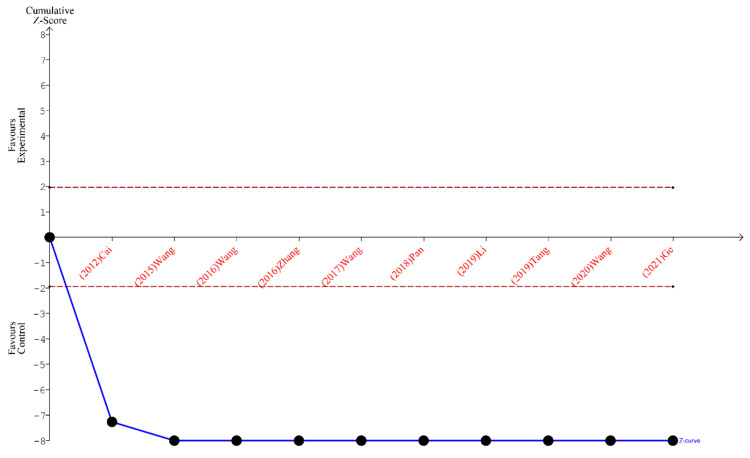
TSA for the articles included in the analysis of the total number of lymph nodes harvested [19,20,21,22,23,24,25,26,27,28].

**Figure 6:**
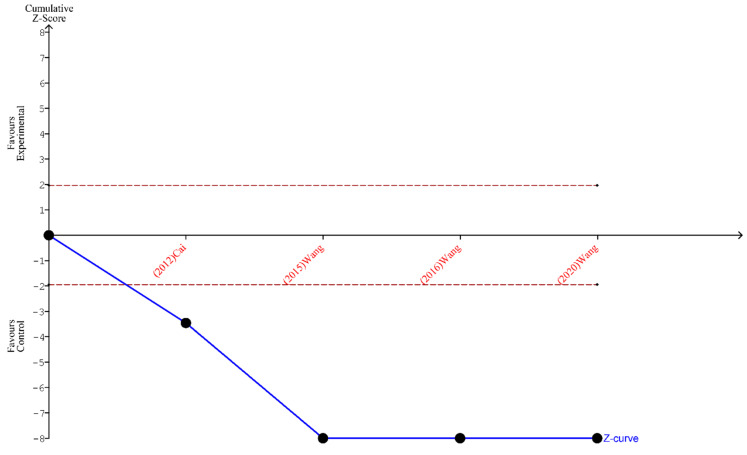
TSA for the articles included in the analysis of the ratio of ≥12 lymph nodes harvested [19,20,21,27].

**Figure 7:**
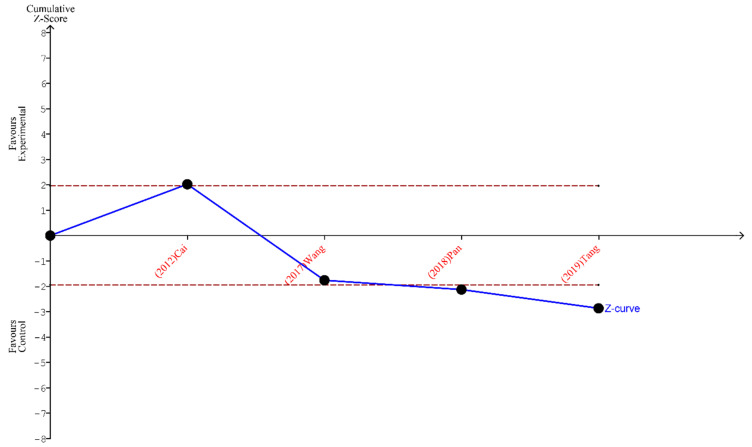
TSA for the articles analyzing the metastatic lymph nodes harvested [19,23,24,26].

Finally, the outcomes of TSA performed for all three analyses indicate that the sample size is sufficient to yield valid results and no future studies on the same subject are needed. These outcomes are shown in Figure 5, Figure 6 and Figure 7.

## 3. Discussion

In this study, we investigated the effectiveness of carbon nanoparticles in lymph node tracing in surgery for colorectal cancer. Our outcomes show that the use of this lymph node tracer results in higher number of lymph nodes harvested during colorectal cancer (6.15 lymph nodes on average), while patients administered carbon nanoparticles also have a higher rate of more than twelve lymph nodes harvested, which is the minimum number of lymph nodes needed for successful staging of colorectal cancer. Nonetheless, the use of carbon nanoparticles does not result in a higher number of metastatic lymph nodes resected, and this can be a result of the fact that carbon nanoparticles do not possess a specific tropism for pathologic lymph nodes. Herein, we document the results of the first systematic review and meta-analysis on the subject that includes studies with different methodologies (retrospective, cross-sectional, prospective, and randomized) and not just randomized control trials.

Cancer theranostics is a novel concept in medicine that focuses on using various metal and carbon nanoparticles in the diagnosis and treatment of malignant diseases [29]. It combines these two fundamental aspects of medicine in a single smart tool, as this multimodal approach gives the opportunity of achieving efficient treatment with an immediate imaging feedback at the same time. In order to achieve this purpose, various theranostics materials have been investigated, such as metallic nanoparticles (e.g., gold, silver, zinc, and iron oxide particles), carbon nanomaterials (e.g., nanodots, nanotubes, graphene, and fullerenes), polymeric assemblies, and rare-earth elements [29]. Lately, carbon nanoparticles have attracted a lot of attention as they possess important properties, such as excellent surface chemistry and high strength, while they also have a wide variety of diversity in their structure (nanodiamond, graphene, carbon nanotubes, carbon nanohorns, and quantum dots) [30]. Until now, carbon-based nanomaterials like graphene oxide and carbon nanodots have received a lot of attention, as they are biodegradable, stable, have excellent photothermal conversion in near-infrared light, and are cost-effective. Nonetheless, the batch-to-batch size and morphological characteristics of graphene oxide nanoparticles are difficult to control; hence, a pragmatic medical application for them presents significant challenges. On the other hand, carbon nanodots are newly discovered 0-D nanomaterials with well-documented surface and size functionalization, enhanced by a good constellation of biological (bioeliminable, biocompatible, and biodegradable) and optical (high near-infrared region photothermic conversion, high-fluorescence quantum yield in the red near-infrared region) that seem to make them superior to graphene oxide nanoparticles, thus paving the way for further research to come up with promising theranostic agents for precision cancer diagnosis and treatment [29]. The carbon nanoparticle suspension was recently authorized by the China Food and Drug Administration, and consists of active nanocarbon combined with physiological saline and polyvinylpyrrolidone [31]. Until now, its administration to humans has not been proven to have any significant adverse outcomes [32].

Nowadays, there are other agents in addition to carbon nanoparticles that are used as lymph node tracers, such as methylene blue, India ink, and indocyanine green (ICG) [33,34,35,36]. Regarding methylene blue, two similar meta-analyses published in 2023 [33,37] concluded that compared to the unstained group, patients who were subjected to methylene blue staining had a higher number of lymph nodes harvested and a higher rate of more than twelve lymph nodes harvested. However, methylene blue has been proven to lead to cardiac anomalies and neurotoxic outcomes in patients that are on serotonergic drugs [38,39]. Regarding India ink, two comparative studies [34,40] indicated that its use is associated with a higher number of lymph nodes harvested, but these findings have not been corroborated by a systematic review or meta-analysis yet. Finally, two meta-analyses on the use of indocyanine green [36,41] showed promising outcomes in terms of sentinel lymph node detection, but poor results overall in detecting metastatic lymph nodes. Nonetheless, there are multiple studies [42,43,44,45,46] that have revealed that the use of indocyanine green results in a decrease in the overall complication rate following colorectal cancer surgery, especially anastomotic leak.

Thus far, there have not been many studies that compare the various lymph node tracing agents to one another in colorectal surgery or test the outcomes of their combined use. Nonetheless, in 2012, Cai et al. published a study on 60 patients that showed that both carbon nanoparticles and methylene blue resulted in a higher number of lymph nodes harvested in colorectal surgery, but there was no superiority identified between the two agents [19]. Moreover, ICG was found to be superior to carbon nanoparticles in lymph node tracing in thyroid cancer surgery, resulting in a higher number of lymph nodes harvested at a lower cost [47]. However, He et al. found similar outcomes between ICG and carbon nanoparticles during surgery for endometrial cancer [48], pointing out that carbon nanoparticles can be used when near-infrared imaging equipment is not available. Another study by Qin et al. showed that the combination of ICG and methylene blue has better results in the detection of sentinel lymph nodes in surgery for breast surgery [49]. Currently, a randomized control study is being carried out to compare ICG and carbon nanoparticles in lymph node tracing in gastric cancer surgery [50]. Nonetheless, currently, data comparing the various lymph node tracers to one another in colorectal surgery are limited. Therefore, specific advantages and disadvantages in the use of one tracer over the other are difficult to identify, and guidelines or suggestions on a specific preference cannot be made at this point. Future comparative studies could shed some more light on this field.

The findings of our study are in agreement with the two previous studies that investigated the role of carbon nanoparticles in colorectal cancer surgery. In their meta-analysis, Li et al. [17] concluded that the use of carbon nanoparticles leads to a higher number of lymph nodes retrieved and a higher ratio of patients with more than twelve lymph nodes harvested. They also concluded that carbon nanoparticles result in an increased detection rate of micro lymph nodes and micro metastatic lymph nodes but not in a higher rate of overall metastatic lymph nodes harvested. More specifically, Li et al. analyzed data originating from 17 randomized control trials with a total number of 1241 patients: 600 in the carbon nanoparticles group and 641 in the control group. The results of the quantitative synthesis of these studies revealed a significantly higher number of lymph nodes retrieved in the carbon nanoparticle group (WMD = 5.21, 95% CI 4.14 to 6.29, *p* < 0.001), while the ratio of patients with more than twelve lymph nodes harvested increased by 14% in the carbon nanoparticles group (RR = 114, 95% CI 1.07 to 1.22, *p* < 0.001). Moreover, this meta-analysis revealed that there was a significant increase in the detection rate of micro lymph nodes (RR = 1.68, 95% CI = 1.38 to 2.04, *p* < 0.001) as well as in the detection rate of micrometastatic lymph nodes (RR = 1.92, 95% CI 1.26 to 2.92, *p* < 0.001) in the carbon nanoparticles group. Nonetheless, the detection rate of metastatic lymph nodes in total was not significantly different (RR = 1.09, 95% CI 0.92 to 1.29, *p* = 0.33), which agrees with the findings of our study. No adverse effects from the use of carbon nanoparticles were reported in any of the studies included in this article. Similarly, Liu et al. [16] conducted a concise review which concluded that the use of carbon nanoparticles results in less intraoperative bleeding, shorter operation time, and shorter time to locate lesions and dissect lymph nodes. Moreover, this study concluded that the use of carbon nanoparticles results in a higher number of lymph nodes harvested and in a higher rate of micro lymph nodes detection. More specifically, Liu et al. analyzed 14 studies (eight randomized control trials, three retrospective studies, and three case–control studies) with a total number of 1618 patients: 887 patients in the carbon nanoparticle infusion group and 731 in the control group. Following analysis, the majority of the studies in the carbon nanoparticle group confirmed that primary tumors as well as lymph nodes could be identified by injecting 0.5–1 mL of carbon nanoparticle suspension into the submucosal stroma in the area of the lesions 10 min to seven days prior to surgery. In two studies, however, submucosal injection in the surrounding tumor area was carried out intraoperatively. Furthermore, in another study evaluating carbon nanoparticles’ feasibility in advanced colorectal cancer, the carbon nanoparticles detection efficiency was assessed eight weeks after chemoradiation therapy (approximately 14 weeks prior to surgery). On top of that, there was also one article that showed that the time of administration had no significant relationship to the efficiency of the technique. Ultimately, thirteen out of the fourteen included studies had reported total and (or) average number of harvested lymph nodes in the carbon nanoparticle group and the control group, respectively. Eleven of these articles revealed that the total (and/or) mean number of identified lymph nodes per patient was significantly increased in the carbon nanoparticle group compared to the control group, highlighting the efficiency of carbon nanoparticles in tracing lymph nodes. Moreover, seven studies recorded the numbers of dissected metastatic lymph nodes, wherein two of them identified a greater number of metastatic lymph nodes in carbon nanoparticles group, while the other four studies revealed no statistically significant difference between the two groups. On top of that, six studies had documented and carried out a comparison of the ratio of patients whose detected lymph nodes were fewer than 12, where two of these studies revealed no statistically significant difference, while the other four of these studies reported a lower ratio of <12 lymph nodes in the carbon nanoparticles group. Nonetheless, by performing TSA for all the comparisons carried out in our study, we conclude that further studies are unlikely to change the outcome of our analysis, in spite of the large heterogeneity among the included studies, and as a result we suggest the use of carbon nanoparticles as lymph node tracers in colorectal cancer on a wider scale. This could potentially lead to better oncological outcomes for patients diagnosed with colorectal cancer, as a higher number of lymph nodes and a higher ratio of successful staging could lead to better postoperative planning of adjuvant treatment, thus increasing the overall survival rate as well as providing a more accurate prognosis. However, the wider use of carbon nanoparticles poses certain challenges. First of all, colorectal surgeons need to receive proper training before becoming able to apply this technique efficiently, and histopathologists also need to be efficient in assessing specimens stained with carbon nanoparticles. Finally, institutions need to be able to cover the cost of acquiring and utilizing these lymph node tracers.

Our study has some limitations, however. First of all, all the included studies originate from China; as a result, it is unclear whether the results can be generalized. This constitutes a potential geographical limit of the results, as medical practice varies globally and different populations respond differently to various treatments. Furthermore, our analysis did not take into account several oncological factors that could affect its outcome, such as tumor histological type, grade, and stage. Also, publication bias has been identified in the first analysis that was performed in our study, while other sources of bias in the included studies could not be identified. As a result, this could lead to further limitations in the interpretation of our results. Finally, the long-term outcomes of the use of carbon nanoparticles have not been investigated; therefore, we cannot draw conclusions regarding the role of carbon nanoparticles in overall survival rate, disease-free survival rate, and recurrence rate of the patients with colorectal cancer. Data on these long-term outcomes are not currently available. Hence, we suggest that future research focuses on these outcomes as they are the most important patient-related outcomes, and if significant differences are identified, then the use of carbon nanoparticles can greatly improve the quality of life of patients with colorectal cancer.

## 4. Materials and Methods

This study is a systematic review and meta-analysis that was conducted in strict accordance with the PRISMA checklist, without applying a registered pre-existing protocol. A detailed and comprehensive online search of the current literature was used to identify articles on the performance of carbon nanoparticles infusion in lymphatic mapping and lymph node dissection during colorectal cancer surgery. The following electronic databases were searched for articles published in English up to 29 April 2023: (1) Medline; (2) Scopus; (3) EMBASE (conference abstracts included); (4) Cochrane Library; and (5) Google Scholar. Additional search for gray literature was carried out on the websites of international surgical, colorectal, and oncological societies, associations, and networks. The following search string was used for searching the online databases: (((colorectal neoplasm*[Title/Abstract]) OR (colorectal cancer[Title/Abstract])) OR (colorectal tumor[Title/Abstract]))) AND (((((nano-carbon[Title/Abstract] OR (carbon particle[Title/Abstract])) OR (carbon nanoparticle[Title/Abstract])) OR (lymph node tracer[Title/Abstract])) OR (lymphatic tracer[Title/Abstract])).

Initially, two independent researchers (G.G. and I.K.) went through the abovementioned databases and assessed the retrieved articles for their eligibility based on the following inclusion criteria: (1) studies performed on human patients; (2) patients diagnosed with colorectal cancer undergoing operative treatment with use of carbon nanoparticles for lymph node tracing (experimental group); (3) patients in the control group who were administered normal saline or not injected prior to surgery; and (4) articles providing sufficient data regarding lymph node dissection and/or lymph node metastases. In any case of disagreement between the two reviewers, a third experienced reviewer (V.A.) was involved and the final decision on the respective articles was made either by consensus or the majority opinion was applied.

Data extraction was carried out by two independent researchers (E.C-W. and A.P.) and the retrieved data were confirmed by another researcher (N.T.). The extracted data of the eligible articles included the names of the authors, the year of publication, sample size of control and experimental groups, and patients’ demographics (age, sex, and cancer type), as well as data on the primary outcomes of the use of carbon nanoparticles (number of retrieved lymph nodes, number of cases of more than 12 lymph nodes harvested, and number of metastatic lymph nodes resected).

In this study, all statistical analyses were carried out using Reviewer Manager 5.4.1 software (Review Manager (RevMan) (computer program), Version 5.4.1, Copenhagen: The Nordic Cochrane Centre, Denmark, The Cochrane Collaboration, 2020) and STATA Version 16.1. Data in this study are presented as mean ± standard deviation, while weighted mean differences (WMDs) and odds ratios (ORs) with a confidence interval (CI) of 95% were calculated for continuous and dichotomous variables, respectively. A *p* value less than 0.05 was used to determine the level of statistical significance. In cases of significant heterogeneity among the studies (I^2^ ≥ 50%), a random effects model was utilized; otherwise, a fixed effects model was applied. The random effects model was selected in order to better control the heterogeneity by assuming that the underlying effects follow a normal distribution. In cases of large heterogeneity, meta-regression analysis was performed to identify whether any residual heterogeneity was present in the analysis of the data. The role of meta-regression analysis was to appropriately combine and contrast multiple subsets of studies in case a single-summary measure did not seem correct or adequate to capture all the clinical or methodological diversity in those subsets. The publication bias was evaluated by designing Begg’s funnel plot and performing the respective Egger’s test. Trial sequential analysis (TSA) was performed using trial sequential analysis (TSA) (computer program), Version 0.9.5.10 Beta, The Copenhagen Trial Unit, Centre for Clinical Intervention Research, The Capital Region, Copenhagen University Hospital—Rigshospitalet, 2021, software in order to assess whether the study sample size was sufficient to reach valid conclusions or if more studies were required. TSA is a recent cumulative meta-analysis method used to weigh type I and II errors and to estimate when the effect is large enough to be unaffected by further studies.

## 5. Conclusions

The objective of this article was to assess the role of carbon nanoparticles as lymph node tracers in colorectal cancer surgery. Our outcomes show that the use of carbon nanoparticles results in an increased number of lymph nodes harvested as well as a higher rate of the minimum number of twelve lymph nodes needed for accurate staging harvested. As a result, we suggest the use of carbon nanoparticles on a wider scale. Further studies will be needed to evaluate the effect of the use of carbon nanoparticles on long-term outcomes in the management of patients with colorectal cancer.

## Figures and Tables

**Table 1:** Characteristics of each individual study included in the meta-analysis (all studies originate from China).

Study	Experimental Group (*n*, Age)	Control Group (*n*, Age)	Sex (Male/Female)	Cancer Type
[19]	20, 57.5 ± 11.5	20, 64.9 ± 7.4	20/20	Colorectal
[20]	45, 53.1 ± 12.0	107, 54.0 ± 11.6	113/39	Rectal
[21]	27, 62.81 ± 11.29	27, 64.63 ± 10.05	33/21	Colorectal
[22]	35, 60.0 ± 10.7	52, 58.9 ± 9.4	40/47	Rectal
[23]	344, 58.6 ± 12.4	126, 59.1 ± 12.2	261/209	Colorectal
[24]	52, not mentioned	47, not mentioned	Not mentioned	Colon
[25]	33, 57.2 ± 9.4	33, 57.8 ± 9.7	52/14	Rectal
[26]	40, 57.9 ± 11.8	39, 59.3 ± 10.7	31/48	Colon
[27]	123, 58.98 ± 9.836	116, 56.78 ± 10.824	146/93	Colorectal
[28]	60, 61.0 ± 11.7	72, 64.2 ± 10.4	81/51	Colorectal

**Table 2:** Mean Difference Inverse-Variance (IV) Random Effects Forest plot with 95% Confidence Interval (CI) of meta-analysis of mean number of lymph nodes harvested.

Study	Experimental			Control			Mean Diff.	Weight	Forest Plot
	n	Mean	sd	n	Mean	sd	IV, Random, 95% CI	(%)	
[19]	20	26.80	8.40	20	12.20	3.20	14.60 [10.66, 18.54]	8.34	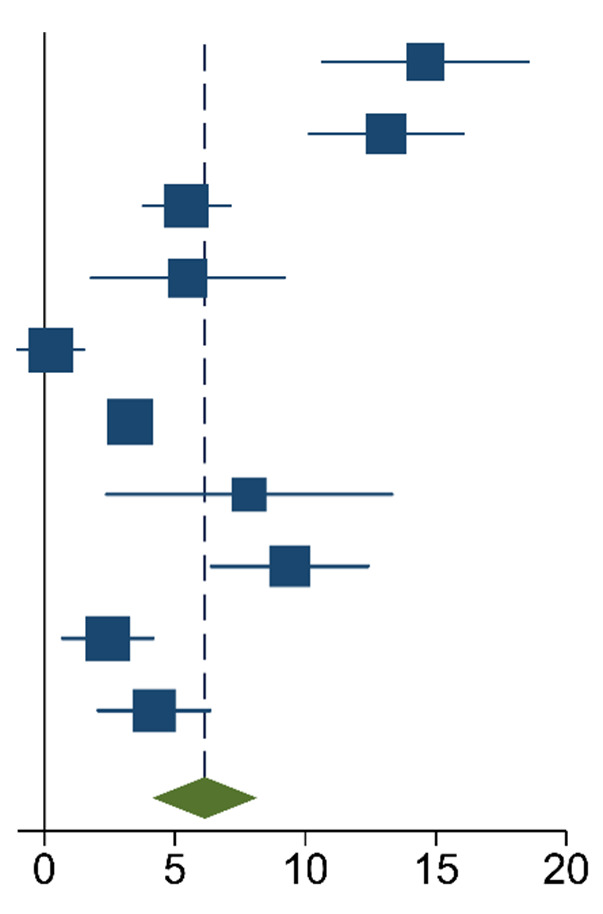
[20]	45	21.10	9.60	107	8.00	4.60	13.10 [10.16, 16.04]	9.72	
[21]	27	14.41	3.32	27	8.96	2.90	5.45 [3.79, 7.11]	11.31	
[22]	35	28.20	9.40	52	22.70	7.30	5.50 [1.81, 9.19]	8.67	
[23]	344	9.13	6.30	126	8.89	6.11	0.24 [−1.02, 1.50]	11.7	
[24]	52	20.90	0.70	47	17.60	0.90	3.30 [2.98, 3.62]	12.23	
[25]	33	24.06	13.20	33	16.21	9.09	7.85 [2.38, 13.32]	6.43	
[26]	40	31.30	8.10	39	21.90	5.30	9.40 [6.39, 12.41]	9.62	
[27]	123	19.84	6.43	116	17.41	7.23	2.43 [0.69, 4.17]	11.23	
[28]	60	19.30	6.70	72	15.10	5.70	4.20 [2.05, 6.35]	10.76	
Total 95% CI	779			639			6.15 [4.14, 8.16]		

Heterogeneity: τ^2^ = 8.57, I^2^ = 92.75%, H^2^ = 13.79. Test of θ_i_ = θ_j_: Q(9) = 124.12, *p* = 0.00. Test of θ = 0: z = 6.00, *p* = 0.00. The vertical solid black line is the no-effect line, and the dashed blue line is the overall effect-size line.

**Table 3:** M-H Random Effects Forest Plot with 95% Confidence Interval (CI) of meta-analysis of ratio of patients with ≥12 lymph nodes harvested.

Study	Experimental		Control		Log Odds-Ratio	Weight	Forest Plot
	Events	Total	Events	Total	IV, Random, 95% CI	(%)	
[19]	20	20	10	20	3.71 [0.78,6.65]	11.68	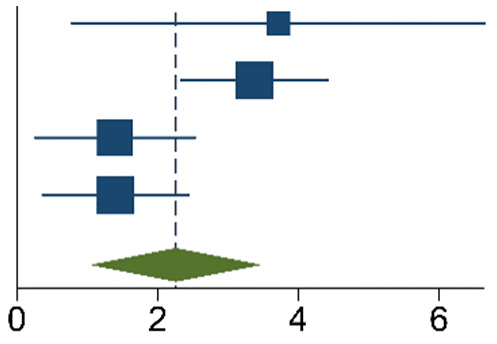
[20]	40	45	23	107	3.37 [2.34,4.41]	29.84	
[21]	19	27	10	27	1.40 [0.26,2.53]	28.57	
[27]	118	123	99	116	1.40 [0.37,2.43]	29.91	
Total					2.26 [1.06,3.46]		

Heterogeneity: τ^2^ = 0.98, I^2^ = 70.48%, H^2^ = 3.39. Test of θ_i_ = θ_j_: Q(3) = 10.16, *p* = 0.02. Test of θ = 0: z = 3.68, *p* = 0.00. The vertical solid black line is the no-effect line, and the dashed blue line is the overall effect-size line.

**Table 4:** M-H Random Effects Forest Plot with 95% Confidence Interval (CI) of meta-analysis of detection rate of metastatic lymph nodes.

Study	Experimental		Control		Log Odds-Ratio	Weight	Forest Plot
	Events	Total	Events	Total	IV, Random, 95% CI	(%)	
[19]	50	535	32	223	−0.49 [−0.96, −0.01]	17.66	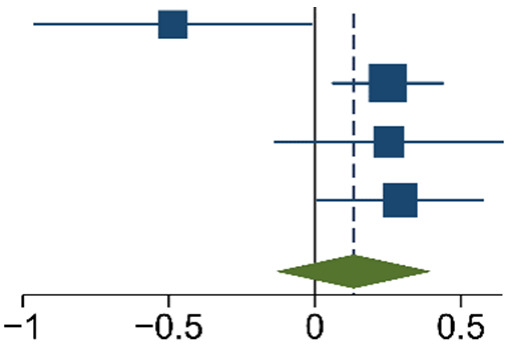
[23]	574	3143	166	1120	0.25 [0.06, 0.44]	33.23	
[24]	72	1088	43	825	0.25 [−0.14, 0.64]	21.59	
[26]	156	1252	82	854	0.29 [0.01, 0.58]	27.52	
Total					0.13 [−0.13,0.40]		

Heterogeneity: τ^2^ = 0.05, I^2^ = 65.85%, H^2^ = 2.93. Test of θ_i_ = θ_j_: Q(3) = 8.79, *p* = 0.03. Test of θ = 0: z = 0.97, *p* = 0.33. The vertical solid black line is the no-effect line, and the dashed blue line is the overall effect-size line.

## Data Availability

The data supporting the findings of this study are available within the article.
